# Optimization of The Cell Aggregates Method for Isolation and Purification of Human Granulosa Cells from Follicular Fluid

**DOI:** 10.22074/ijfs.2020.5790

**Published:** 2019-11-11

**Authors:** Georges Raad, Marwa Bazzi, Judy Tanios, Youmna Mourad, Joan Azouri, Joseph Azouri, Chadi Fakih

**Affiliations:** 1 Azoury IVF Clinic, Mount Lebanon Hospital, Camille Chamoun Bvd, Beirut, Lebanon; 2 Al-Hadi Laboratory and Medical Center, Beirut, Lebanon; 3 Lebanese University, Faculty of Sciences, Section II, Fanar, Lebanon and Azoury IVF Clinic, Mount Lebanon Hospital, Camille Chamoun Bvd, Beirut, Lebanon; 4 OB-GYN Department, Inova Fairfax Hospital, Falls Church, Virginia

**Keywords:** Density Gradient, Follicular Fluid, Granulosa Cells, Isolation and Purification

## Abstract

**Background:**

Aspirated ovarian follicular fluids (FF) contain luteal granulosa cells (LGCs) and other contaminating cell types. Several strategies, such as the antibody binding methods, the flask method, the cell strainer and positive selection of granulosa aggregates after density gradient (DG) centrifugation, were tested as LGC purification methods. Each of these strategies has its own advantages and disadvantages. Positive selection of granulosa aggregates after DG centrifugation is simple, rapid and efficient in terms of LGC recovery. However, it results in a low purity. Here, we aimed to test whether modifying the traditional protocol by collecting the aggregates from the FF, before the DG centrifugation could decrease the percentage of contaminating cells.

**Materials and Methods:**

In the present prospective study, 32 FF, from 32 women,were randomly assigned into one of the two purification techniques: positive selection of granulosa aggregates from the FF, after DG centrifugation (DG/ Agg, n=16) or positive selection of granulosa aggregates from the FF, before DG centrifugation (Agg/DG, n=16). At the end of each procedure cell count, vitality, morphology and purity of the cell suspension were evaluated.

**Results:**

No significant difference was detected in the total number of GCs between DG/Agg and Agg/DG (P>0.05). However, higher percentage of GCs with normal morphology was detected in Agg/DG compared to DG/Agg (P<0.001). Moreover, lower percentages of white blood cells (P<0.01), red blood cells (P<0.001) and epithelial cells (P<0.01) were identified in Agg/DG compared to DG/Agg.

**Conclusion:**

Here we showed that positive selection of granulosa aggregates from the FF prior to DG technique had a higher purity compared to the traditional protocol. Thus, it could be a method of choice to prepare GCs for research purposes in clinical *in vitro* fertilization settings.

## Introduction

Granulosa cells (GCs) are the somatic cells surrounding the oocyte in the ovary (1). A bi-directional communication is set between GCs and the oocyte via locally secreted factors (2, 3). This cross-talk plays an important role in the differentiation of the GCs and the oocyte (2). In addition, GCs secrete sex hormones (e.g. estrogen and progesterone) under the control of the endocrine system to regulate the function of several body organs (4). After ovulation, GCs become luteinized (LGCs) and secrete progesterone to support potential pregnancy (5). Altogether, these characteristics made from LGCs an interesting model to study the ovarian physiology (5, 6).

In assisted reproductive technology, GCs can be collected from follicular fluid (FF) during oocyte retrieval, form women undergoing controlled ovarian stimulation (COS) (5). The GCs in FF may be present as free cells or as clearly visible aggregates (Aggs). In parallel, other cell types could also be detected in this fluid, such as white blood cells, red blood cells and epithelial cells (7). Therefore, different strategies are used to separate LGCs from other FF contaminants (8-10).

The efficiency of purification methods that are based on the differential physical properties of LGCs and contaminting cells were tested in several reports (5, 8, 10). Positive selection of granulosa Aggs after density gradient (DG) procedure, under a dissecting microscope, is among the tested strategies (5, 7). It is a rapid, simple and relatively inexpensive technique (7). In addition, it allows the recovery of high LGC percentage (5, 7). However, it retains a certain percentage of contaminating cells. This limits the reliability of the results of some subsequent techniques, such as quantitative polymerase chain reaction (qPCR) and RNA chains analysis (5).

Therefore, the aim of present study was to test whether isolating granulosa Aggs at the beginning of purification procedure would decrease the percentage of contaminating cells at the DG interface. In order to answer this biological question, we collected the granulosa Aggs (which are larger than other FF contaminants) directly from the FF and then subjected them to the DG centrifugation. Next, we compared the outcome of this modified protocol to that of traditional one. This comparison was performed in terms of the percentage of recovered LGC, vitality and purity.

## Materials and Methods

### Collection of luteal granulosa cells

FFs were collected from preovulatory follicles of young women (<38 years old) undergoing oocytes retrieval for intra-cytoplasmic sperm injection (ICSI), via transvaginal ultrasound-guided aspiration (n=32) (5). After cumulus-oocyte complex (COC) collection from the FF for ICSI, the remaining liquid was directly assigned for LGC collection (within no longer than 5 minutes) (11). Before proceeding with oocytes retrieval, these women underwent COS. It was made up of gonadotropin-releasing hormone (GnRH) antagonist for pituitary down-regulation and recombinant follicle stimulating hormone (FSH) for ovarian stimulation. When three follicles reached 16 mm in diameter, subcutaneous injection of recombinant human chorionic gonadotropin (hCG) was given for ovulation induction (12). It is important to note that we excluded all women with poor ovarian response from the study, according to Bologna criteria (13). Couples gave their written informed consent and the study protocol form was approved by Mount Lebanon hospital Ethical Committee (MLH code: OBS2018-002). 

### Experimental design

**Fig 1:**
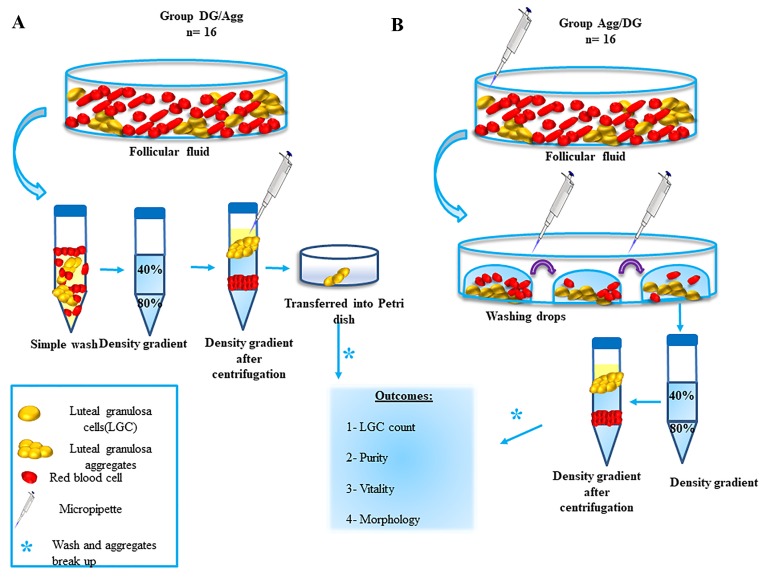
The schematic of luteal GCs purification procedures. A. Follicular fluid samples (n=16) were subjected to a simple wash, then subjected to a two layered (40-80%) density gradient centrifugation. After that, the luteal Aggs were transferred into a petri dish using a micropipette, washed and dissociated. Finally, LGC count, purity, vitality and morphology were assessed. B. Using a micropipette, luteal Aggs were collected from the FF samples (n=16), moved into washing drops to reduce contamination and then subjected to a two layered (40-80%) density gradient centrifugation. After that, the Aggs were washed, dissociated and analysed in terms of LGC count, purity, vitality and morphology. GCs; Granulosa cells, Aggs; Granulosa cell aggregates, FF; Follicular fluids, LGCs; Luteal GCs, and DG; Density gradient.

COC-free FFs (n=32) were randomly assigned to one of two IVF GC preparation methods. The first technique was positive selection of granulosa Aggs, after DG centrifugation (DG): DG/Agg (Fig .1A) (5, 7) . The second technique was a positive selection of granulosa Aggs directly from FF, before DG centrifugation: Agg/DG (Fig .1B). Each technique was performed on 16 samples from 16 women. At the end of both preparation methods, total cell concentration was estimated, percentage of total cell vitality was established, and purity of the obtained cell suspension was evaluated (Fig .1) (5). It is important to note that all of the centrifugation steps were performed using an Eppendorf 5702 centrifuge (Eppendorf, Lebanon).

### Tested luteal granulosa cell preparation methods

#### Positive selection of granulosa aggregates, after density gradient centrifugation

Each FF was pooled into a 14 ml falcon tube and centrifuged for 10 minutes at 2000 rpm. The collected pellet was gently pipetted onto a DG made up of two layers: 40% and 80% (Sperm Gradient Kit, Sydney IVF, COOK medical, EMEC Lebanon). After centrifugation for 10 minutes at 1200 rpm, the ring-like layer at the interface was transferred into a 60 mm petri dish. The Aggs were positively selected under a dissecting microscope and washed in human tubal fluid medium (HTF medium, Life Global, Ibra Haddad Lebanon). The wash consisted of a centrifugation for 10 minutes at 2000 rpm. Next, the pellet was resuspended in 1 ml HTF. Then, Aggs breaking up was performed mechanically, using a Pasteur pipette (Fig .1A) (5, 7).

#### Positive selection of granulosa aggregates from the follicular fluid, before density gradient centrifugation 

Aggregates were collected from the COC-free FF, in HTF medium. Next, these Aggs were gently pipetted onto a DG made up of two layers: 40% and 80%. They were next centrifuged for 10 minutes at 1200 rpm. The ring-like layer in the interface was then transferred into a 5 ml round tube, mixed with 1 ml HTF and centrifuged for 10 minutes at 2000 rpm. At the end, the pellet was suspended in 1 ml HTF followed by up- and down-pipetting for 1 minute, to dissociate the Aggs (Fig .1B).

#### Estimation of total cell count and viability

The purified GCs were counted using a hemocytometer microscopic slide and cell viability was determined using Trypan Blue (0.4%) (14-18).

### Evaluation of the cell suspension purity and luteal granulosa cells morphology using Wright-Giemsa stain

**Fig 2:**
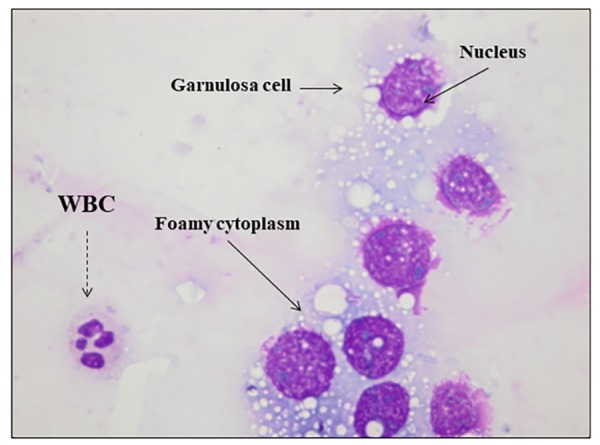
A cluster of granulosa cells stained with Wright stain. A cluster of granulosa cells (GCs) stained with Wright stain. The GC is made up of a central nucleus (purple) and foamy cytoplasm (clear purple): solid arrows. A white blood cell (WBC, multi-lobed nucleus) was observed: dashed arrow (magnification: ×100 under immersion oil).

In order to compare purity of the cell suspension and the LGCs morphology derived from DG/Agg and Agg/DG techniques, a thin smear slide was prepared from each cell suspension (19). The smears were stained with WrightGiemsa (8, 19). The interpretation of cytological slides was evaluated by a clinical pathologist who was blinded to the used technique. A cytologically normal GC is characterized by a large dark-stained nucleus, a foamy paler cytoplasm and intact cell having no cytoplasmic shrinkage (Fig .2) (19, 20). By contrast, the neutrophils were distinguished by their multi-lobed nucleus, lymphocytes had a nucleus occupying most of the cell volume, eosinophils contained a bilobed nucleus, and the monocytes had about 20 µm diameter with an irregular nucleus (11). Moreover, the total granulosa count was estimated by: (% of granulosa on Wright-Giemsa smear) X (total cell count on the hemocytometer microscopic slide).

### Statistical analysis

Statistical analysis was performed using IBM SPSS 23 software (IBM SPSS Statistics for Windows, Version 23.0. Armonk, NY: IBM Corp). Data with normal distribution were then compared using independent samples t test. Data with non-normal distribution were compared using the Mann-Whitney non-parametric test. All data were presented as median [interquartile range (IQR)]. Categorical data were compared using Chi-square test. Results were considered statistically significant for a P<0.05.

## Results

### Population characteristics

There was no statistically significant difference in the female age (P=0.5), number of retrieved oocytes (P=0.2) and infertility etiology (P=0.8) between these two techniques (Table 1).

**Table 1:** Comparison of the population characteristics in DG/Agg and Agg/DG groups


Population characteristics		DG/Agg technique	Agg/DG technique	P value

Infertility etiology	Female age (Y)	32.25 ± 5.4	31.06 ± 5.35	0.5
	Number of retrieved oocytes	9 ± 6.23	12 ± 6.10	0.2
	Male factor (%)	6/16 (37.5)	5/16 (31.3)	0.8
	Female factor (%)	4/16 (25)	6/16 (37.5)	
	Male and female factors (%)	2/16 (12.5)	1/16 (6.3)	
	Unexplained infertility (%)	4/16 (25)	4/16 (25)	


Data are presented as mean ± SD or n (%).

Results are expressed as mean ± standard deviation (SD) for normally distributed continuous variables and percentage for categorical data. Continuous variables were compared using the independent samples t test. Categorical data were compared using the Chi-square statistical test. There were no statistically significant difference between the groups of female age (P=0.5), number of retrieved oocytes (P=0.2) and infertility etiology (P=0.8). P>0.05 indicates that there is no statistically significant difference between two groups. 

### Assessment of cell concentration and vitality between DG/Agg and Agg/DG techniques

**Fig 3:**
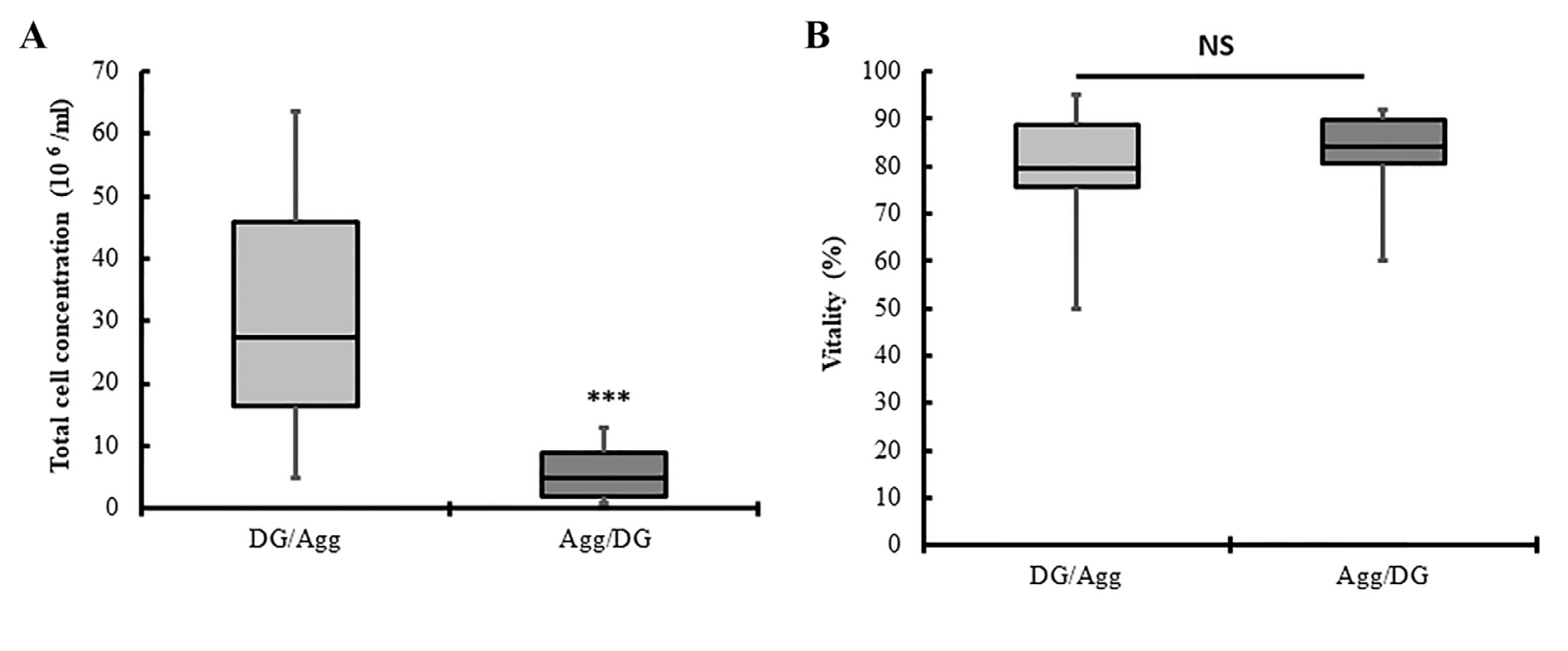
Boxplots show the total cell concentration and vitality percentage in DG/Agg and Agg/DG techniques. Boxes indicate the interquartile range. Horizontal bars within the boxes indicate the median. Whiskers indicate the range of data. Data were compared using the Mann-Whitney non-parametric test. A. Boxplot shows a lower concentration of all cell types after Agg/DG technique compared to DG/Agg technique (***; P<0.001). B. Boxplots show no statistically significant difference in the vitality percentage of cells after two techniques. NS; No significant difference.

Hemocytometer slide and trypan blue staining were used to assess total cell concentration and vitality after two purification techniques. In one hand, a significantly lower concentration of cells was obtained after Agg/DG compared to DG/Agg (P<0.001, Fig .3A). On the other hand, no significant difference was detected in the vitality percentage between DG/Agg and Agg/DG (P>0.05, Fig .3B). 

### Evaluation of the granulosa percentage, total count and morphology between the two techniques

**Fig 4:**
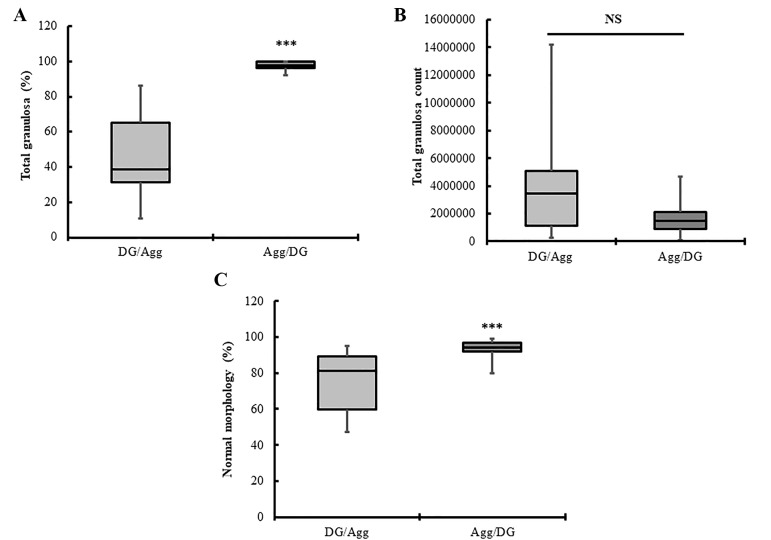
Boxplots show total recovered granulosa and percentage of GCs with normal morphology in DG/Agg and Agg/DG techniques. Boxes indicate the interquartile range. Horizontal bars within boxes indicate the median. Whiskers indicate the range of data. Data were compared using the Mann-Whitney non-parametric test. A. Boxplot shows a higher percentage of total GCs after Agg/DG compared to DG/Agg (***; P<0.01), B. Boxplots show that there was no statistically significant difference in the total granulosa count after DG/Agg and Agg/DG techniques. C. Boxplots show a higher percentage of GCs with normal morphology in the Agg/ DG group companed to the DG/Agg group. NS; No significant difference.

A thin smear was prepared from each cell suspension, after processing and they were stained using WrightGiemsa stain (Fig .2). A significant higher percentage of granulosa was identified in the cell suspension after Agg/ DG compared to DG/Agg (P<0.001, Fig .4A). Moreover, no significant difference was detected in the total granulosa count between the two techniques (P>0.05, Fig .4B). Of particular interest, the percentage of granulosa with normal morphology was significantly higher post-Agg/ DG compared to DG/Agg (P<0.001, Fig .4C). 

### Estimation of cell suspension purity in the two purification techniques

**Fig 5:**
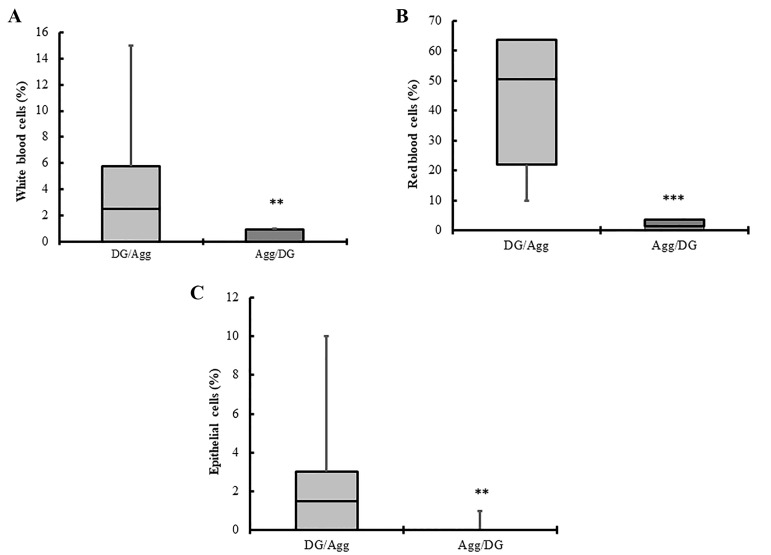
Boxplots show the percentage of contaminant cell types presented after DG/Agg and Agg/DG techniques. Boxes indicate the interquartile range. Horizontal bars within boxes indicate the median. Whiskers indi- cate the range of data. Data were compared using the Mann-Whitney non-parametric test. A. Boxplots show a lower percentage of white blood cells in the Agg/DG technique compared to the DG/Agg technique (**; P<0.01). B. Boxplots show a lower percentage of red blood cells in the Agg/DG technique compared to DG/Agg technique (***; P<0.001). C. Box- plots show a lower percentage of epithelial cells in the Agg/DG technique compared to the DG/Agg technique (**; P<0.01).

Purity of each preparation was estimated on WrightGiemsa stained smears (Fig .2). Compared to DG/Agg, significantly lower percentages of white blood cells (P<0.01, Fig .5A), red blood cells (P<0.001, Fig .5B) and epithelial cells (P<0.01, Fig .5C) were detected in Agg/ DG technique.

## Discussion

The aim of present report was to decrease percentages of contaminating cells in the suspension obtained from the positive selection of granulosa Aggs after DG procedure. Here we showed that collecting Aggs from the FF prior to DG centrifugation significantly decreased the percentages of contaminating cells.

In assisted reproductive technology, aspirated human FF contains heterogeneous population of cells (10). For instance, it contains LGCs that could be collected for research purposes (8). It also comprises white blood cells which play an important role in the process of ovulation (21, 22). In addition, it could be contaminated by red blood cells and epithelial cells originating from the invasive trans-vaginal guided-aspiration (5, 10).

Studying quality, quantity and gene expression of the GCs may improve the information given about ovarian function and oocyte physiology (23-25). Therefore, scientists have tested several strategies to extract LGCs from the FF.

The purification strategies that are based on the immunorecognition of specific cell markers, such as fluorescence activated cell sorting (FACS), magnetic activating cell sorting (MACS), and Dynabeads, are considered to be the most efficient in terms of purity and less efficient in LGCs recovery (5, 10, 26-28). However, besides achieving a certain level of purity, it is of paramount importance to maintain cell count, vitality and morphology during purification procedures in order to perform subsequent investigations (10, 29). Here, comes the positive selection of granulosa Aggs after DG centrifugation, which is characterized by its simplicity, affordability, high speed of operation and efficiency to recover a high number of LGCs (5, 7). This technique depends on the large size of Aggs allowing the ease of their identification in FF (7). Another purification method -the flask method- takes advantage of the ability of immune cells to adhere to plastic ware, while GCs remain in suspension. However, selecting granulosa Aggs from FF after DG or collecting them using the flask method could not efficiently isolate GCs from other FF contaminants. 

Interestingly, our proposed protocol (positive selection of granulosa Aggs before DG centrifugation) led to a lower contamination by red blood cells, white blood cells and epithelial cells. Actually isolating granulosa Aggs from the heterogeneous cell population at the beginning of the purification procedure could explain the lower level of contamination obtained using this modified protocol. It is essential to mention that application of a cell strainer to collect GCs can also reduce the level of white blood cells despite being more expensive than our proposed procedure (5).

In addition, our modified protocol resulted in a vitality percentage as high as the original procedure (positive selection of granulose Aggs after density gradient). Strikingly however, the percentage of granulosa with normal morphology was higher in the suspension obtained from our modified procedure compared to the original procedure. In fact, GCs are very sensitive to reactive oxygen species (ROS) (30). ROS production could be increased due to the activation of leukocytes during centrifugation (31-33). The positive selection of granulosa Aggs from FF after DG centrifugation comprises two centrifugation steps during which ROS producing leukocytes are still in contact with GCs. This could have affected the GCs thus resulting in a lower percentage cells with normal morphology.

## Conclusion

The positive selection of GCs before subjecting them to a DG centrifugation surpassed the original procedure in terms of purity and recovery of granulosa with normal morphology. It resulted in a relatively high number of recovered LGCs less contaminated by other cell types. In addition to its efficiency, the modified protocol is simple, inexpensive and rapidly operated. That being the case, it could be a method of choice to prepare GCs for research purposes in clinical settings *in vitro*.
